# From brain circuits to self-regulation: an integrative neurocognitive model of metacognition and emotion regulation in ADHD

**DOI:** 10.3389/fpsyt.2026.1830766

**Published:** 2026-07-31

**Authors:** Davoud Amiri, Lamberto Briziarelli, Swetang J. Shah, Sara Amiri, Amin Vakili, Amir Hossein Jalali Nadoushan

**Affiliations:** 1 Specialist in Psychiatry and Social Medicine, Uppsala Region, Uppsala, Sweden; 2 Department of Public Health, Faculty of Medicine, University of Perugia, Perugia, Italy; 3 Clinical Director, Bay Area Community Health (BACH), San Jose, CA, United States; Adjunct Clinical Associate Professor, AT Still University, San Jose, CA, United States; 4 Department of Economics, Uppsala University, Uppsala, Sweden; 5 Specialist in Psychiatry, Amicus Medical Chambers, Brisbane, QLD, Australia; 6 Mental Health Research Center, School of Medicine, Iran University of Medical Sciences, Tehran, Iran

**Keywords:** attention-deficit/hyperactivity disorder (ADHD), cognitive control, emotion regulation, fronto-limbic networks, fronto-striatal circuits, metacognition, self-regulation

## Abstract

**Background:**

Attention-deficit/hyperactivity disorder (ADHD) has traditionally been conceptualised as a disorder of attention and executive functioning. However, increasing evidence indicates that emotional dysregulation is highly prevalent across the lifespan and contributes substantially to functional impairment. Neuroimaging and neurochemical studies implicate abnormalities in fronto-striatal and fronto-limbic networks, including the dorsolateral prefrontal cortex, anterior cingulate cortex, amygdala, and ventral striatum. Despite these advances, existing theoretical frameworks rarely integrate neurobiological mechanisms, metacognitive control processes, emotion regulation, and clinical symptom expression within a single explanatory framework.

**Objective:**

This narrative review aims to synthesise evidence across neuroscience, cognitive psychology, and clinical psychiatry to propose an integrative neurocognitive model linking brain circuitry, metacognitive control, emotion regulation, and behavioural manifestations in ADHD.

**Methods:**

Relevant literature published between 1995 and 2025 was identified through searches of PubMed, Scopus, and Web of Science using combinations of terms related to ADHD, emotion regulation, metacognition, executive function, dopamine, glutamate, and fronto-striatal and fronto-limbic circuitry. Evidence was synthesised across four interconnected domains: neurobiological mechanisms, metacognitive control processes, emotion regulation, and clinical ADHD phenotype.

**Results:**

Converging evidence suggests that dysfunction within prefrontal regulatory systems may impair metacognitive monitoring and top-down control. Reduced prefrontal regulation of limbic structures, particularly the amygdala and ventral striatum, may increase emotional reactivity and impulsive behavioural responses. These interacting mechanisms provide a plausible pathway linking neural circuit dysfunction with emotional dysregulation and core behavioural manifestations of ADHD.

**Conclusion:**

ADHD may be conceptualised as a disorder of hierarchical self-regulation in which disturbances in neural circuitry cascade through metacognitive and emotional regulatory systems to produce characteristic behavioural symptoms. This integrative framework may inform future research and support the development of mechanism-based interventions targeting self-regulatory processes in ADHD.

## Introduction

Attention-deficit/hyperactivity disorder (ADHD) is a common neurodevelopmental condition characterised by persistent patterns of inattention, impulsivity, and hyperactivity that interfere with academic, occupational, and social functioning. Although ADHD has traditionally been conceptualised primarily as a disorder of attention and executive functioning, accumulating evidence suggests that emotional dysregulation represents a clinically significant dimension of the disorder in many individuals, particularly when functional impairment and comorbidity are considered (1). Emotional dysregulation in ADHD often manifests as heightened emotional reactivity, frustration intolerance, rapid shifts in affect, and difficulty modulating emotional responses in everyday situations.

These emotional difficulties are associated with substantial impairments in interpersonal relationships, educational performance, occupational functioning, and overall quality of life. Nevertheless, emotional processes have historically received less emphasis in theoretical models of ADHD compared with cognitive and behavioural symptoms such as attentional deficits and inhibitory control problems.

Advances in neuroscience have begun to clarify the neural mechanisms underlying these regulatory disturbances. Neuroimaging and neurochemical studies consistently implicate alterations in fronto-striatal and fronto-limbic circuitry, including the dorsolateral prefrontal cortex (dlPFC), anterior cingulate cortex (ACC), amygdala, and ventral striatum. These interconnected networks are critically involved in reward processing, cognitive control, emotional evaluation, and behavioural regulation. Dysfunctions within these circuits have been linked to abnormalities in dopaminergic and glutamatergic signalling, suggesting that ADHD may involve disturbances in neural systems responsible for adaptive self-regulation (2–5).

At the cognitive level, impairments in metacognitive control processes may represent a key bridge between neural dysfunction and behavioural symptoms. Metacognition refers to the capacity to monitor, evaluate, and regulate one’s own cognitive processes, including self-monitoring, goal maintenance, and conflict detection. Deficits in these processes may weaken top-down control mechanisms that normally regulate emotional responses and behavioural impulses.

Despite growing evidence linking neurobiology, cognitive control, and emotional dysregulation in ADHD, relatively few theoretical frameworks have attempted to integrate these domains within a unified explanatory model. Most existing studies examine isolated components of the disorder, such as executive functioning, reward processing, or emotional reactivity, without explicitly addressing how these levels interact within a broader regulatory system.

The aim of the present narrative review is therefore to synthesise evidence across four interconnected levels of analysis: neurobiological mechanisms, metacognitive control processes, emotion regulation, and the clinical phenotype of ADHD. By integrating findings across neuroscience, cognitive psychology, and clinical psychiatry, we propose a multilevel neurocognitive model in which impaired metacognitive regulation within prefrontal networks contributes to reduced top-down control over limbic emotional systems. This interaction may amplify impulsivity, emotional instability, and behavioural dysregulation characteristic of ADHD.

The proposed model is not intended to replace previous executive-function, reward-based, or self-regulation accounts of ADHD (6, 7). Rather, it aims to organise selected elements of these frameworks within a clinically oriented multilevel model linking neural circuitry, metacognitive monitoring, emotion regulation, and behavioural manifestations. Its main contribution is therefore integrative rather than competing: it attempts to clarify how established neurobiological and cognitive mechanisms may interact across levels of self-regulation and contribute to clinically observable symptoms.

Such an integrative perspective may contribute to a more comprehensive understanding of ADHD and inform the development of mechanism-based assessment and intervention strategies targeting self-regulatory processes.

## Methods

### Study design

This study was conducted as a narrative review aiming to integrate evidence from neuroscience, cognitive psychology, and clinical psychiatry regarding the relationships between neurobiological mechanisms, metacognitive control processes, emotion regulation, and behavioural manifestations in attention-deficit/hyperactivity disorder (ADHD).

A narrative review approach was chosen because the objective of the study was conceptual integration across multiple domains rather than quantitative synthesis of effect sizes.

### Literature search strategy

A structured literature search was conducted in three electronic databases:

- PubMed
- Web of Science
- Scopus

Articles published between 1995 and 2025 were considered.

Search terms were combined using Boolean operators and included keywords related to ADHD, neurobiology, metacognition, and emotional regulation. The following terms were used:

- ADHD
- attention-deficit/hyperactivity disorder
- emotion regulation
- emotional dysregulation
- metacognition
- cognitive control
- executive function
- self-regulation
- dopamine
- glutamate
- fronto-striatal circuits
- fronto-limbic networks
- prefrontal cortex
- anterior cingulate cortex
- amygdala
- reward processing

An example search query was:

ADHD AND (emotion regulation OR emotional dysregulation) AND (metacognition OR cognitive control OR executive function) AND (prefrontal cortex OR amygdala OR striatum OR dopamine OR glutamate OR fronto-striatal OR fronto-limbic)

### Study selection

Studies were included if they addressed at least one of the following domains:

1. Neurobiological mechanisms of ADHD (e.g., dopaminergic signalling, glutamatergic neurotransmission, fronto-striatal and fronto-limbic circuitry)
2. Metacognitive and executive control processes in ADHD (e.g., self-monitoring, cognitive control, conflict monitoring)
3. Emotion regulation in ADHD (e.g., emotional reactivity, affective impulsivity, amygdala–prefrontal regulation)
4. Clinical manifestations of ADHD (e.g., impulsivity, executive dysfunction, emotional instability)

Priority was given to:

- systematic reviews
- meta-analyses
- large neuroimaging studies
- longitudinal or experimental studies
- highly cited theoretical papers relevant to ADHD self-regulation models.

### Data synthesis

Because the primary aim of the review was conceptual integration, findings were synthesised thematically rather than quantitatively.

Evidence was organised across four interconnected levels of analysis:

1. Neurobiological mechanisms
2. Metacognitive control processes
3. Emotion regulation
4. Clinical ADHD phenotype

The goal of the synthesis was to identify converging mechanisms across these domains and to develop an integrative neurocognitive framework linking neural circuitry, metacognitive regulation, emotional processes, and behavioural symptoms in ADHD.

### Conceptual model development

Based on the integrated literature, a multilevel neurocognitive model was developed describing how disturbances in fronto-striatal and fronto-limbic networks may impair metacognitive monitoring and top-down cognitive control. Reduced prefrontal regulation of limbic systems may subsequently contribute to emotional dysregulation and impulsive behaviour, ultimately shaping the clinical ADHD phenotype.

This review did not aim to perform a formal systematic review or meta-analysis but rather to integrate converging evidence across multiple scientific disciplines to develop a conceptual model of ADHD self-regulation.

## Results

### Overview of the literature

The literature search identified a large body of research examining neurobiological mechanisms, executive and metacognitive processes, and emotional regulation in ADHD. Evidence from neuroimaging, neuropsychology, and clinical research converges on the idea that ADHD involves disturbances in neural systems supporting self-regulation.

Across the reviewed studies, findings clustered around four interconnected domains:

1. Neurobiological mechanisms
2. Metacognitive and cognitive control processes
3. Emotion regulation mechanisms
4. Clinical manifestations of ADHD

These domains together form the basis of a multilevel framework linking neural circuitry with behavioural symptoms. **Table 1** summarises the main neurocognitive domains relevant to self-regulation in ADHD.

**Table 1:** Summary of evidence across neurocognitive domains relevant to self-regulation in ADHD.

Domain	Key mechanisms	Representative evidence	Main limitations	Relevance to proposed model
Neurobiological mechanisms	Altered fronto-striatal and fronto-limbic circuitry; dysregulation of dopaminergic and glutamatergic signalling; altered reward anticipation and prefrontal regulatory control	Neuroimaging and neurochemical studies implicate the dlPFC, ACC, amygdala, and ventral striatum in cognitive control, reward processing, emotional evaluation, and behavioural regulation	Findings vary across age groups, medication status, comorbidity, imaging methods, and task paradigms. Most studies examine isolated circuits rather than multilevel regulatory systems	Provides the neural foundation for impaired self-regulation and reduced top-down modulation of emotional and behavioural responses
Metacognition and cognitive control	Impaired self-monitoring, goal maintenance, planning, conflict detection, working memory, inhibition, and cognitive flexibility	Executive-function and metacognitive models suggest that ADHD involves difficulties monitoring and regulating one’s own cognitive processes	Metacognition is variably defined and measured. Few studies directly connect metacognitive impairment with neural circuitry and emotion regulation in the same design	Represents the cognitive bridge between neural circuit dysfunction and behavioural symptoms
Emotion regulation	Heightened emotional reactivity; affective impulsivity; reduced cognitive reappraisal; altered amygdala–prefrontal regulation	Clinical and neuroimaging studies indicate that emotional dysregulation is common in ADHD and may be associated with altered prefrontal–limbic connectivity	Emotional dysregulation is not included as a core diagnostic criterion and is measured inconsistently across studies. It is also difficult to separate from comorbid anxiety, depression, trauma, or mood instability	Explains how reduced regulatory control may translate into emotional lability, frustration intolerance, and impulsive reactions
Clinical ADHD phenotype	Inattention, impulsivity, hyperactivity, executive dysfunction, emotional instability, and functional impairment	Clinical studies show that ADHD symptoms often co-occur with emotional and self-regulatory difficulties across academic, occupational, and interpersonal domains	ADHD is heterogeneous. Subtypes, developmental stage, sex, medication status, trauma exposure, and comorbidities may modify symptom expression	Provides the observable clinical level of the model, where neural, cognitive, and emotional dysregulation become functionally relevant
Developmental and heterogeneity factors	Delayed or inefficient maturation of prefrontal control systems; earlier maturation of limbic and reward systems; variation by age, subtype, and comorbidity	Developmental models suggest imbalance between reward/emotional reactivity and prefrontal regulatory control, particularly during adolescence	Longitudinal and multimodal studies remain limited. Many studies do not adequately stratify by ADHD presentation, age, sex, or comorbidity	Prevents overgeneralisation by showing that the model should be interpreted as a flexible framework rather than a single pathway for all ADHD presentations

A. Neurobiology of ADHD

*(Brain circuits and neurotransmitter systems)*

Neuroimaging and neurochemical studies consistently implicate abnormalities within fronto-striatal and fronto-limbic networks in ADHD (8–10). These networks include the dorsolateral prefrontal cortex (dlPFC), anterior cingulate cortex (ACC), amygdala, and ventral striatum. These regions collectively support executive control, reward processing, emotional evaluation, and behavioural regulation.

Meta-analytic evidence indicates altered activity within the ventral striatum during reward anticipation, suggesting dysregulation of dopaminergic reward pathways (2, 11, 12).

Neurochemical studies further suggest that glutamatergic signalling within fronto-striatal circuits may also be altered in ADHD, potentially affecting excitatory communication between cortical and subcortical regions (4, 13).

Functional connectivity studies demonstrate altered interactions between prefrontal regulatory regions and limbic structures such as the amygdala, particularly in individuals with pronounced emotional dysregulation (14, 15).

These findings collectively suggest that ADHD involves disruptions in neural systems responsible for integrating cognitive control, emotional processing, and reward-based motivation.

**Figure 1:**
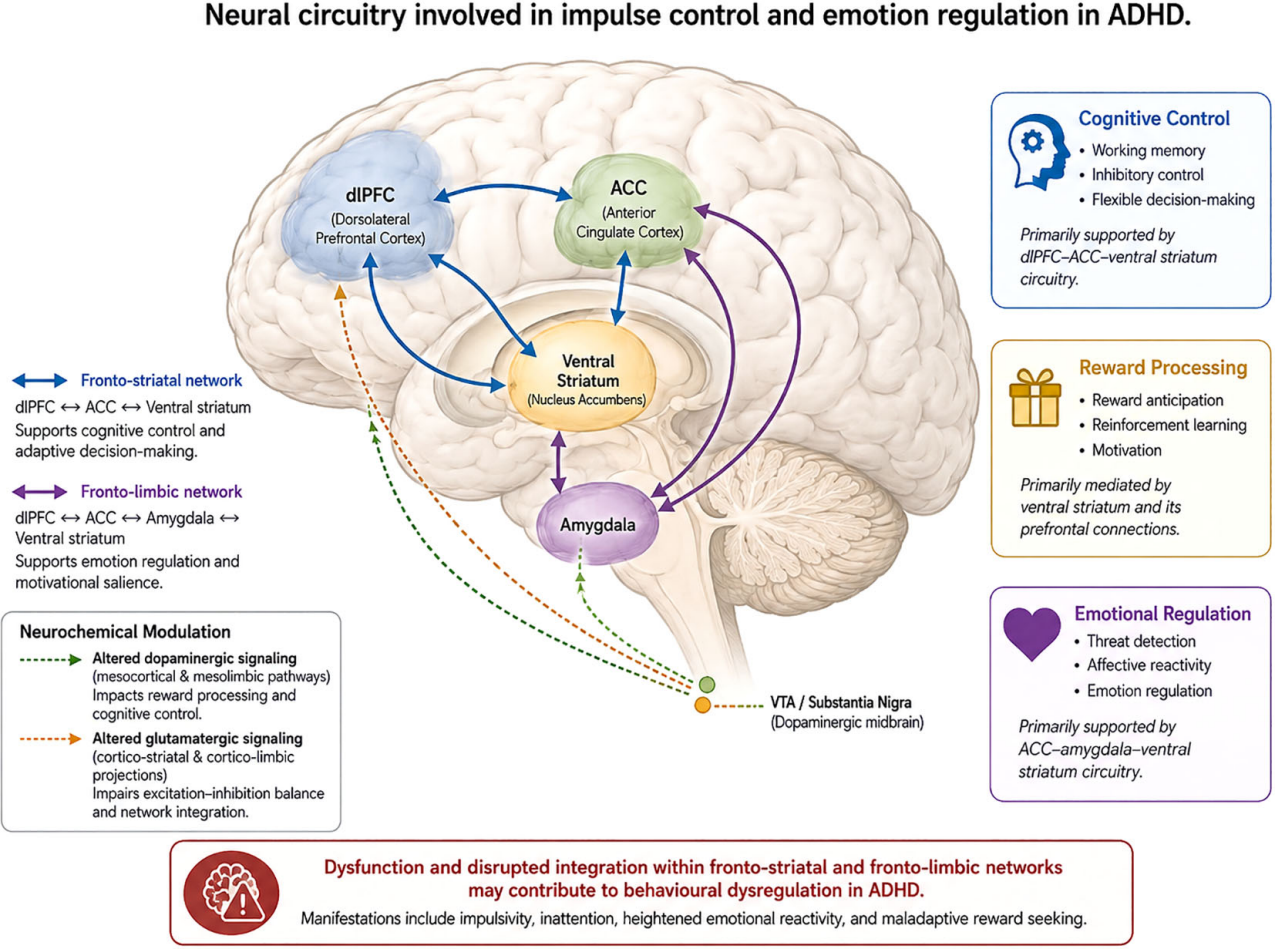
Neural circuitry involved in impulse control and emotion regulation in ADHD.

**Figure 1** illustrates the proposed interaction between fronto-striatal and fronto-limbic networks involved in cognitive control, reward processing, and emotion regulation in ADHD. Dysfunction within circuits involving the dorsolateral prefrontal cortex, anterior cingulate cortex, amygdala, and ventral striatum may disrupt the integration of executive control, motivational salience, and emotional regulation. Alterations in dopaminergic and glutamatergic signalling within these networks may further contribute to impaired behavioural regulation, impulsivity, emotional reactivity, and maladaptive reward-seeking behaviour.

B. Metacognition and cognitive control

(Executive monitoring and awareness processes)

At the cognitive level, individuals with ADHD frequently demonstrate impairments in executive functioning and metacognitive monitoring. Executive functions such as working memory, inhibitory control, and cognitive flexibility depend heavily on prefrontal cortical networks, particularly the dlPFC and ACC (16–18).

Metacognition refers to the capacity to monitor and regulate one’s own cognitive processes, including planning, self-evaluation, and behavioural adjustment. Evidence suggests that deficits in self-monitoring and goal maintenance may weaken top-down regulatory control in ADHD (19–21).

Impairments in behavioural inhibition and executive control have long been proposed as central mechanisms underlying ADHD symptoms (19, 20, 22). Developmental research suggests that executive functions continue to mature throughout childhood and adolescence, contributing to improvements in self-regulation across development (23, 24).

Metacognitive interventions targeting self-monitoring and awareness processes have shown promise in adults with ADHD (25, 26).

Neurocognitive models increasingly suggest that ADHD extends beyond a narrow account of executive dysfunction and involves broader disturbances in cognition and regulatory control (27).

In the present model, metacognition is not treated as a separate mechanism independent of executive functioning, but rather as a higher-order aspect of self-regulation that depends on executive processes such as goal maintenance, error monitoring, inhibitory control, and cognitive flexibility.

C. Emotion Regulation in ADHD

(Amygdala–prefrontal regulation and emotional impulsivity)

Emotion dysregulation has increasingly been recognised as a clinically important dimension of ADHD, although it is not included as a core diagnostic criterion and should not be interpreted as a universal feature of the disorder (1, 28, 29). Many individuals with ADHD experience heightened emotional reactivity, rapid mood shifts, and difficulty modulating affective responses in everyday situations.

Executive dysfunction and working-memory deficits may further contribute to difficulties regulating emotional responses (30, 31). Emotional dysregulation has therefore been proposed as an important dimension of ADHD that extends beyond traditional cognitive symptoms (32, 33).

Neuroimaging studies suggest that these emotional difficulties may arise from altered functional connectivity between the amygdala and prefrontal regulatory systems. Increased amygdala reactivity and reduced prefrontal inhibition have been associated with emotional lability and impulsive responses in ADHD (14, 15, 34).

Behavioural studies examining cognitive reappraisal and expressive suppression strategies indicate that individuals with ADHD may rely less on adaptive emotion regulation strategies, contributing to difficulties controlling emotional responses (35, 36).

Together, these findings support the hypothesis that impaired top-down regulation from prefrontal networks may permit stronger influence of limbic emotional systems, thereby increasing emotional impulsivity and affective instability in ADHD.

D. Clinical ADHD phenotype

(Behavioural expression and symptom dimensions)

The behavioural manifestations of ADHD may be understood as the observable consequences of disturbances across neural, cognitive, and emotional regulatory systems.

At the clinical level, ADHD is characterised by patterns of inattention, impulsivity, hyperactivity, and executive dysfunction (22, 37–39). However, emotional instability and frustration intolerance are also commonly reported and contribute significantly to functional impairment across academic, occupational, and interpersonal domains. Rather than representing independent symptom clusters, these behavioural features may reflect different expressions of a broader disturbance in hierarchical self-regulation.

### Integrated neurocognitive model of ADHD

Synthesising evidence across these domains suggests that ADHD may involve cascading disturbances across multiple levels of self-regulation.

**Table 2** operationalises the proposed model by linking each theoretical component to testable hypotheses, measurable variables, and possible empirical approaches. This framework is not intended to imply a single linear pathway for all individuals with ADHD. Rather, it provides a structure for future studies to examine how neural circuitry, metacognitive control, emotion regulation, and clinical symptoms interact dynamically across development and across heterogeneous ADHD presentations.

**Table 2:** Operationalisation of the proposed neurocognitive model: testable hypotheses and measurable variables.

Model component	Testable hypothesis	Measurable variables	Possible empirical approach	Clinical relevance
Fronto-striatal and fronto-limbic circuitry	Altered functional connectivity between prefrontal regulatory regions and limbic/reward-related structures will be associated with greater emotional dysregulation and impulsivity in ADHD	Functional connectivity between dlPFC, ACC, amygdala, and ventral striatum; reward anticipation response; emotional reactivity scores; impulsivity measures	fMRI during emotional regulation, reward anticipation, or inhibitory control tasks; resting-state connectivity analyses	May identify neurobiological markers of impaired self-regulation and guide mechanism-based treatment selection
Metacognitive control	Impaired self-monitoring and goal maintenance will mediate the association between prefrontal regulatory dysfunction and behavioural ADHD symptoms	Metacognitive awareness scales; executive-function ratings; working memory; conflict monitoring; error awareness; goal-maintenance tasks	Behavioural testing combined with neuroimaging or ecological momentary assessment	May support the use of metacognitive training and structured self-monitoring interventions
Emotion regulation	Reduced top-down control over limbic emotional responses will predict emotional lability, frustration intolerance, and affective impulsivity	Emotion regulation questionnaires; affective lability scales; amygdala reactivity; prefrontal–amygdala connectivity; reappraisal and suppression measures	Emotional reactivity tasks; longitudinal assessment of emotional symptoms; treatment-response studies	Supports systematic assessment of emotional dysregulation as a clinically relevant ADHD dimension
Behavioural ADHD phenotype	Clinical symptoms of inattention, impulsivity, and emotional instability will reflect combined disturbances across neural, metacognitive, and emotional regulatory levels	ADHD symptom scales; functional impairment ratings; impulsivity measures; occupational, academic, and interpersonal functioning	Multimodal clinical studies combining symptom ratings, cognitive testing, and neurobiological measures	Helps explain why patients with similar ADHD diagnoses may show different functional impairments and treatment needs
Developmental and heterogeneity factors	Age, ADHD presentation, sex, medication status, trauma exposure, and psychiatric comorbidity will moderate the relationship between neural circuitry, metacognition, emotion regulation, and clinical symptoms	Age group; ADHD presentation; comorbid anxiety, depression, autism traits, trauma exposure; medication status; developmental stage	Longitudinal cohort studies; stratified analyses; moderator analyses; developmental neuroimaging	Reduces overgeneralisation and supports personalised, developmentally informed assessment and intervention

At the neural level, dysregulation within fronto-striatal and fronto-limbic circuits may weaken prefrontal regulatory control. These neural alterations may impair metacognitive monitoring processes responsible for maintaining awareness of goals and regulating behaviour.

Reduced metacognitive control may in turn weaken top-down regulation of limbic emotional systems, allowing increased emotional reactivity and impulsive behavioural responses. These interacting mechanisms ultimately manifest as the clinical symptoms of ADHD.

**Figure 2:**
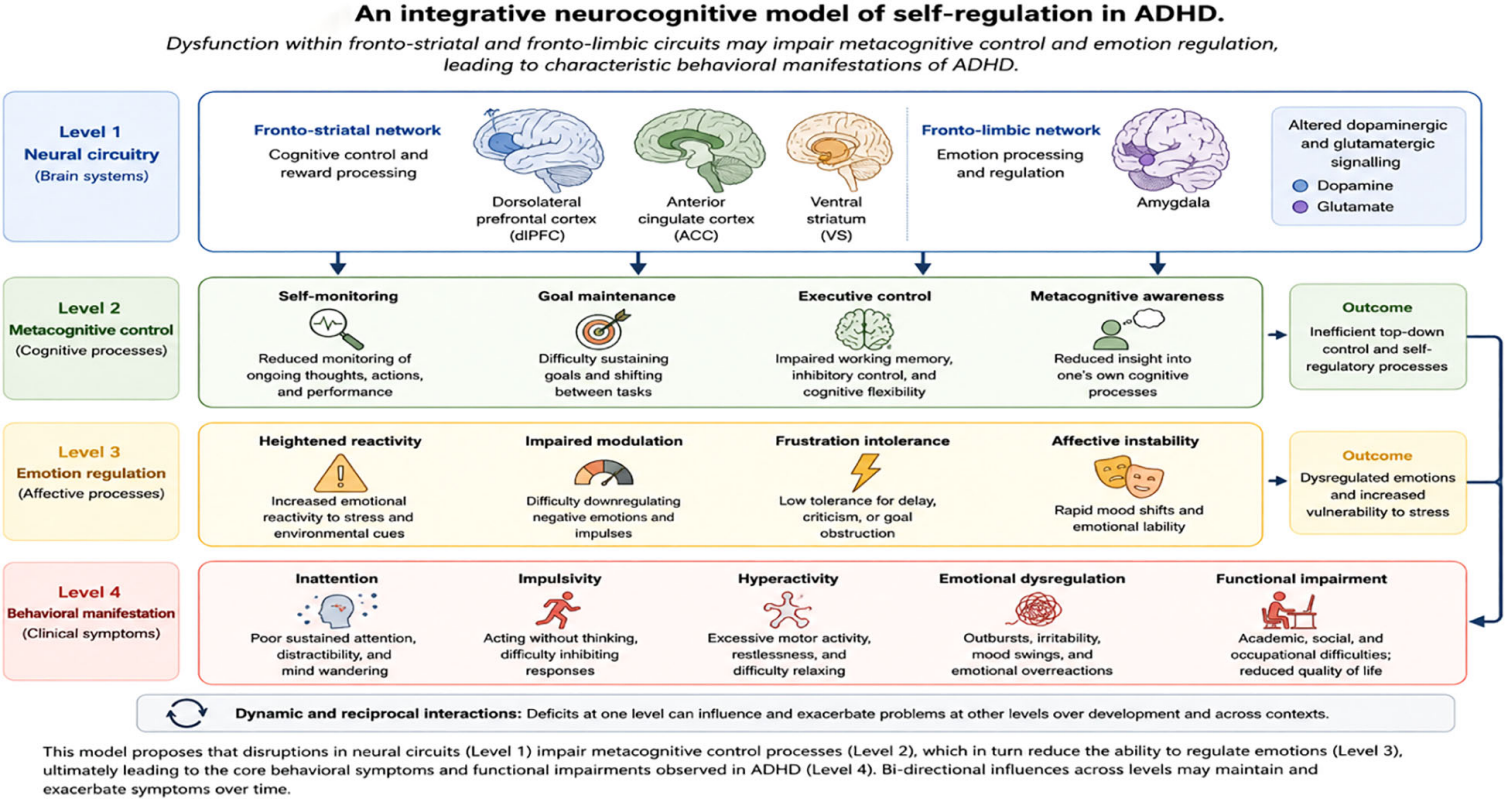
An integrative neurocognitive model of self-regulation in ADHD.

**Figure 2** illustrates the proposed hierarchical and reciprocal framework linking neural circuitry, metacognitive control, emotion regulation, and behavioural manifestations in ADHD. The model emphasises that dysfunction within fronto-striatal and fronto-limbic circuits may impair metacognitive monitoring and top-down regulation, which in turn may contribute to emotional dysregulation and behavioural symptoms. Importantly, the model should not be interpreted as a simple linear pathway; rather, bidirectional and feedback-based interactions across neural, cognitive, emotional, and behavioural levels may maintain or exacerbate symptoms over time.

### Developmental perspective on self-regulation in ADHD

Evidence from developmental neuroscience suggests that regulatory systems supporting cognitive control mature gradually across childhood and adolescence. During early development, limbic and reward-related systems may mature earlier than prefrontal control networks, creating a temporary imbalance between emotional reactivity and cognitive regulation.

In individuals with ADHD, this imbalance may be amplified, contributing to heightened reward sensitivity, impulsivity, and emotional instability during adolescence. Persistence of these regulatory difficulties into adulthood may reflect delayed or inefficient maturation of prefrontal control systems.

**Figure 3:**
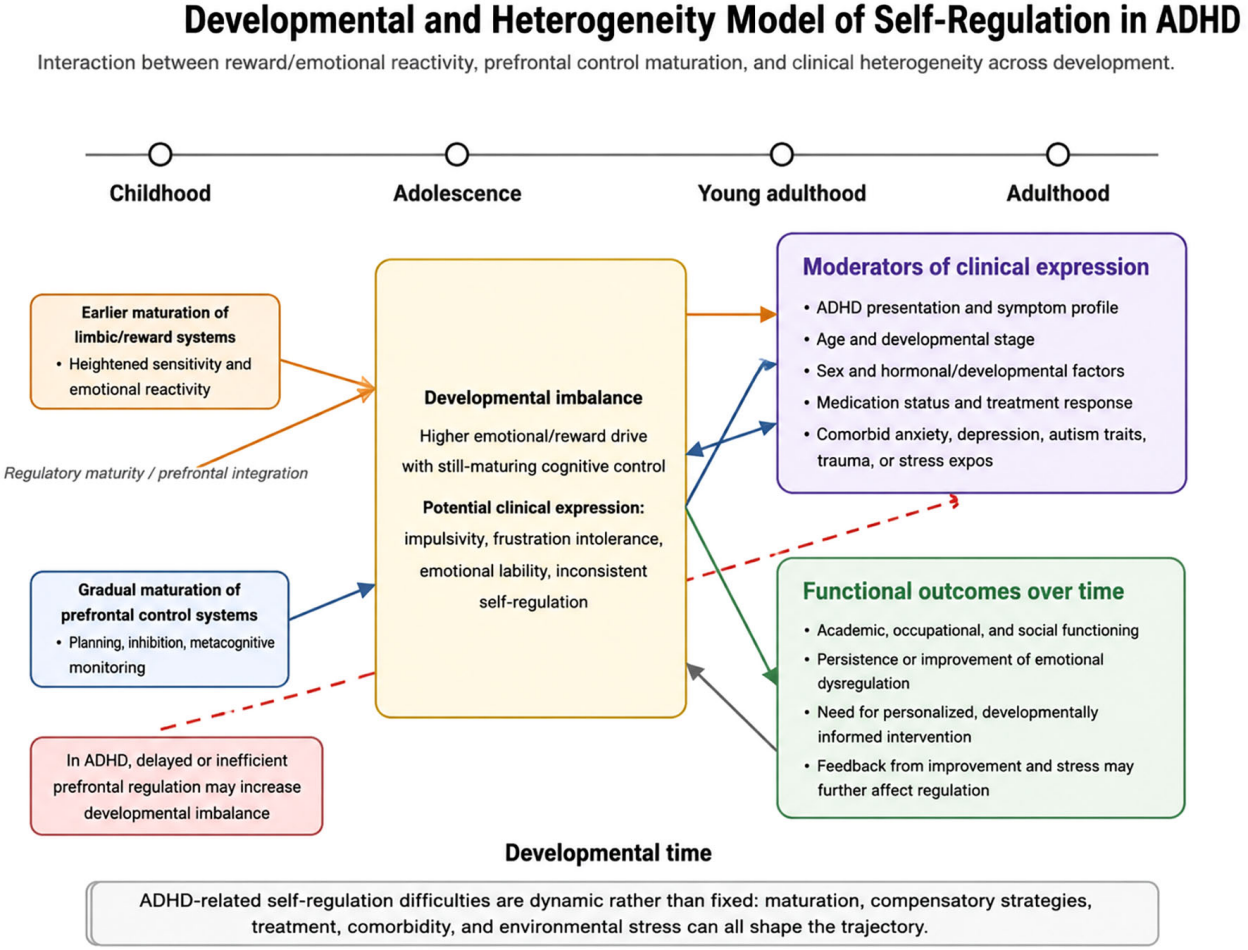
Developmental and heterogeneity model of self-regulation in ADHD.

**Figure 3** illustrates how developmental imbalance between earlier-maturing limbic and reward-related systems and later-maturing prefrontal regulatory systems may contribute to impaired self-regulation in ADHD. The model also highlights that ADHD-related outcomes are not uniform, but may be moderated by age, ADHD presentation, sex, medication status, comorbidity, trauma exposure, stress, and environmental demands. These developmental and heterogeneity factors may influence the expression of impulsivity, emotional dysregulation, functional impairment, and treatment response across the lifespan.

## Discussion

The present review aimed to integrate evidence from neuroscience, cognitive psychology, and clinical psychiatry to propose a multilevel neurocognitive framework linking brain circuitry, metacognitive control, emotion regulation, and behavioural manifestations in attention-deficit/hyperactivity disorder (ADHD). The synthesis of the literature suggests that ADHD may be conceptualised as a disorder of hierarchical self-regulation in which disturbances in neural circuitry cascade through cognitive and emotional regulatory systems to produce the characteristic clinical phenotype.

### Integration of neurobiology, metacognition, and emotion regulation

Accumulating evidence indicates that ADHD is associated with alterations in fronto-striatal and fronto-limbic networks, including the dorsolateral prefrontal cortex, anterior cingulate cortex, amygdala, and ventral striatum. These circuits are critically involved in cognitive control, reward processing, and emotional evaluation. Dysregulation within these systems has been linked to abnormalities in dopaminergic and glutamatergic signalling, as well as stress-sensitive impairment of prefrontal cortical regulation (5), suggesting disturbances in neural mechanisms responsible for adaptive behavioural regulation.

At the cognitive level, impairments in metacognitive control processes may represent an important intermediary mechanism linking neural dysfunction with behavioural symptoms. Metacognition encompasses the capacity to monitor and regulate one’s own cognitive processes, including self-monitoring, conflict detection, and goal maintenance. Inefficient functioning within prefrontal regulatory systems may weaken these monitoring processes, reducing the ability to exert top-down control over behaviour and emotional responses.

Emotion regulation difficulties appear to represent a critical interface between cognitive control mechanisms and observable clinical symptoms. Evidence from neuroimaging studies indicates altered functional connectivity between the amygdala and prefrontal regulatory regions in individuals with ADHD. Reduced prefrontal inhibition of limbic structures may allow increased emotional reactivity and impulsive behavioural responses, contributing to emotional lability and frustration intolerance frequently observed in clinical populations.

### A hierarchical model of self-regulation in ADHD

The integrative model proposed in this review suggests that ADHD symptoms may arise from disruptions across multiple regulatory levels. At the neurobiological level, abnormalities in fronto-striatal and fronto-limbic circuits may impair the functioning of prefrontal control systems. These disturbances may weaken metacognitive monitoring processes responsible for maintaining behavioural goals and regulating responses to environmental demands.

Reduced metacognitive regulation may subsequently impair the ability of prefrontal networks to modulate limbic emotional systems. As a result, emotional responses may become more reactive and less subject to cognitive regulation. These processes ultimately manifest as the behavioural symptoms characteristic of ADHD, including impulsivity, executive dysfunction, and emotional instability.

Conceptualising ADHD as a disorder of hierarchical self-regulation may help integrate previously separate lines of research focusing on executive functioning, reward processing, and emotional dysregulation. Rather than representing independent mechanisms, these domains may reflect different levels of a broader regulatory system spanning neural, cognitive, and behavioural processes.

### Developmental considerations

Developmental neuroscience provides additional insight into the mechanisms underlying ADHD. Prefrontal regulatory systems responsible for executive control and metacognitive monitoring continue to mature throughout adolescence and early adulthood. In contrast, limbic and reward-related systems may develop earlier, potentially creating a developmental imbalance between emotional reactivity and cognitive control.

In individuals with ADHD, this imbalance may be amplified due to altered maturation of fronto-striatal and fronto-limbic networks. Heightened reward sensitivity and emotional reactivity combined with immature cognitive control systems may contribute to impulsive behaviour and emotional instability during adolescence. Persistence of these regulatory difficulties into adulthood may reflect delayed or inefficient maturation of prefrontal control mechanisms.

### Clinical implications

Understanding ADHD as a disorder of hierarchical self-regulation has several implications for clinical assessment and intervention. First, it highlights the importance of evaluating emotional regulation difficulties alongside traditional measures of attentional and executive functioning. Emotional dysregulation may represent a clinically meaningful dimension of ADHD that contributes substantially to functional impairment.

Pharmacological treatments targeting catecholaminergic systems remain among the most effective interventions for ADHD (40).

Second, interventions targeting metacognitive and self-regulatory processes may complement existing pharmacological treatments. Approaches focusing on self-monitoring, cognitive control training, and awareness of emotional states may strengthen top-down regulatory mechanisms and improve behavioural outcomes.

Finally, integrating neurobiological and cognitive perspectives may facilitate the development of mechanism-based treatment strategies that target specific regulatory processes rather than isolated symptom clusters.

### Critical appraisal and unresolved issues

Although converging evidence supports links between fronto-striatal and fronto-limbic circuitry, metacognitive control, and emotion regulation in ADHD, several uncertainties remain. First, the literature is heterogeneous with regard to age, medication status, comorbidity, task design, imaging methods, and definitions of emotional dysregulation. Second, many studies examine isolated mechanisms rather than testing neural, cognitive, emotional, and behavioural processes within the same design. Third, the directionality of the proposed relationships remains insufficiently established, as most available studies are cross-sectional. Finally, emotional dysregulation is measured inconsistently and may overlap with comorbid anxiety, depression, trauma-related symptoms, or autism traits.

These limitations mean that the proposed model should be interpreted as an integrative and hypothesis-generating framework rather than as a confirmed causal pathway. Future longitudinal, multimodal, and intervention-based studies are required to test whether changes in neural circuitry, metacognitive control, and emotion regulation predict clinical outcomes and treatment response in ADHD.

### Limitations

Several limitations should be considered when interpreting the findings of this review. First, the present study employed a narrative review methodology rather than a formal systematic review or meta-analysis. Consequently, the synthesis reflects conceptual integration of the literature rather than quantitative evaluation of effect sizes.

Second, although substantial evidence exists for each component of the proposed framework, relatively few studies simultaneously examine neurobiological mechanisms, metacognitive control processes, and emotional regulation within the same experimental design. Future research integrating these levels of analysis may provide stronger empirical validation of the proposed model.

Third, heterogeneity across ADHD populations in terms of age, comorbid conditions, and symptom presentation may influence the generalisability of the findings.

### Future research directions

Future research should aim to examine the interactions between neural circuitry, metacognitive processes, and emotional regulation using multimodal approaches combining neuroimaging, behavioural assessment, and longitudinal designs. Investigating how these systems interact across development may provide important insights into the emergence and persistence of ADHD symptoms.

Additionally, intervention studies examining the effects of metacognitive training, cognitive control interventions, and emotion regulation strategies may help clarify whether strengthening regulatory mechanisms can improve clinical outcomes in individuals with ADHD.

## Conclusion

The present review proposes an integrative neurocognitive model linking neural circuitry, metacognitive control processes, emotion regulation, and behavioural symptoms in attention-deficit/hyperactivity disorder (ADHD). Evidence from neuroscience, cognitive psychology, and clinical research suggests that disturbances within fronto-striatal and fronto-limbic networks may impair metacognitive monitoring and top-down cognitive control, thereby weakening prefrontal regulation of limbic emotional systems. These interacting mechanisms may contribute to the impulsivity, executive dysfunction, and emotional instability commonly observed in ADHD.

Conceptualising ADHD as a disorder of hierarchical self-regulation may offer a more comprehensive framework for understanding the disorder by integrating neural, cognitive, and emotional processes within a unified model. Such a perspective provides a framework for future research examining interactions between neural circuitry, metacognitive regulation, and emotional functioning in ADHD.
